# Impact of “Green Revolution” gene *Rht-B1b* on coleoptile length of wheat

**DOI:** 10.3389/fpls.2023.1147019

**Published:** 2023-03-02

**Authors:** Dengan Xu, Qianlin Hao, Tingzhi Yang, Xinru Lv, Huimin Qin, Yalin Wang, Chenfei Jia, Wenxing Liu, Xuehuan Dai, Jianbin Zeng, Hongsheng Zhang, Zhonghu He, Xianchun Xia, Shuanghe Cao, Wujun Ma

**Affiliations:** ^1^ College of Agronomy, Qingdao Agricultural University, Qingdao, China; ^2^ National Wheat Improvement Center, Institute of Crop Science, Chinese Academy of Agricultural Sciences, Beijing, China

**Keywords:** wheat, coleoptile length, *Rht-B1b*, QTL, transcriptome

## Abstract

Wheat coleoptile is a sheath-like structure that helps to deliver the first leaf from embryo to the soil surface. Here, a RIL population consisting of 245 lines derived from Zhou 8425B × Chinese Spring cross was genotyped by the high-density Illumina iSelect 90K assay for coleoptile length (CL) QTL mapping. Three QTL for CL were mapped on chromosomes 2BL, 4BS and 4DS. Of them, two major QTL *QCL.qau-4BS* and *QCL.qau-4DS* were detected, which could explain 9.1%–22.2% of the phenotypic variances across environments on *Rht-B1* and *Rht-D1* loci, respectively. Several studies have reported that *Rht-B1b* may reduce the length of wheat CL but no study has been carried out at molecular level. In order to verify that the *Rht-B1* gene is the functional gene for the 4B QTL, an overexpression line *Rht-B1b*-OE and a CRISPR/SpCas9 line *Rht-B1b*-KO were studied. The results showed that *Rht-B1b* overexpression could reduce the CL, while loss-of-function of *Rht-B1b* would increase the CL relative to that of the null transgenic plants (TNL). To dissect the underlying regulatory mechanism of *Rht-B1b* on CL, comparative RNA-Seq was conducted between *Rht-B1b*-OE and TNL. Transcriptome profiles revealed a few key pathways involving the function of *Rht-B1b* in coleoptile development, including phytohormones, circadian rhythm and starch and sucrose metabolism. Our findings may facilitate wheat breeding for longer coleoptiles to improve seedling early vigor for better penetration through the soil crust in arid regions.

## Introduction

Wheat (*Triticum aestivum* L.) is one of the most important food crops in the world, providing large amounts of starch, rich protein and dietary fiber for humans (Asseng et al., 2020). Maintenance of high and stable wheat yields is crucial for global food security (Boyer, 2004). Drought is an important abiotic stress seriously limiting wheat production (Gupta et al., 2020). Arid and semi-arid regions account for about 60% of global crop production, and drought stress caused by frequent extreme weather events often leads to severe reduction of wheat production (Puttamadanayaka et al., 2020). To ensure the emergence rate under drought stress, deeper sowing is often adopted for better utilization of the water in soil (Zhao et al., 2022). However, a sowing depth beyond the coleoptile length (CL) will result in poor stand establishment, late emergence, and slow early leaf development (BR, 1976; Schillinger et al., 1998). Wheat coleoptiles facilitate the stem and the first leaf to break the ground, and directly determine the maximum sowing depth (Rebetzke et al., 2007a; Rebetzke et al., 2014). However, the short coleoptiles of modern semi-dwarf wheat varieties reduce emergence when sown deep (Zhao et al., 2022). Understanding the genetic basis for CL will help developing high-yield semi-dwarf varieties with longer coleoptiles and suitable for deep sowing. Previous studies have demonstrated that CL has high heritability and additive effects and is controlled by multiple genes (Rebetzke et al., 2004; Rebetzke et al., 2007b). Hence, it is feasible to increase the CL through genetic manipulation.

In the 1960s and 1970s, the wide application of dwarf genes *Rht1* (*Rht-B1b*) and *Rht2* (*Rht-D1b*) combined with the increased application of chemical fertilizer greatly promoted the increase of wheat yield, which was called the “Green Revolution” of wheat (Peng et al., 1999; Hedden, 2003). However, compared with the wild type *Rht-B1a*, the dwarf gene, while improving the resistance to colonization and harvesting index, led to increased nitrogen fertilizer requirement, decreased 1000-grain weight, lower grain protein content, drought tolerance, lower anthers exposure rate and susceptibility to scab (Tang et al., 2009; Lanning et al., 2012; Zhang et al., 2013; He et al., 2016). Genetic analysis has also predicted that *Rht-B1b* and *Rht-D1b* loci have certain shortening effects on the CL of wheat, but there has been no further evidence for this speculation (Ellis et al., 2004; Rebetzke et al., 2007b; Yu and Bai, 2010; Li et al., 2011). In contrast, other two widely used *Rht* genes, *Rht8* and *Rht24*, have been proved to have no negative effect on CL, providing an opportunity to breed semi-dwarfing wheat cultivars with long coleoptiles (Würschum et al., 2017; Chai et al., 2022; Tian et al., 2022; Xiong et al., 2022).

Here, we demonstrated that *Rht-B1* is the functional gene underlying a CL QTL on chromosome 4B and its dwarfing allele (*Rht-B1b*) reduces the CL through multiple pathways such as phytohormones, circadian rhythm and starch and sucrose metabolism. These results provide valuable information for wheat breeding of longer coleoptiles to improve the seedling early vigor and penetration through soil crust in arid regions.

## Materials and methods

### Plant materials and phenotyping

A total of 245 F_2:10_ RILs derived from the cross of Zhou 8425B × Chinese Spring were used in this study. Zhou 8425B (Pedigree: Zhou 78A/Annong 7959) and Chinese Spring are an elite facultative wheat line and Chinese landrace, respectively. Zhou 8425B contains two dwarfing alleles *Rht-B1b* and *Rht-D1b* and has a short coleoptile length (CL) of about 3.3 cm. Chinese Spring contains two wildtype alleles *Rht-B1a* and *Rht-D1a*, which contribute to a long CL of about 4.8 cm. Seeds were sampled from plants grown and harvested at Shijiazhuang of Hebei Province and Qingdao of Shandong Province during the 2020–2021 and 2021–2022 cropping seasons, respectively. Good-quality seeds without any visible damage were selected for all lines. Seeds of all parental and progeny lines were sown in cylindrical pots (100 mm high and 80 mm in diameter) at a sowing depth of 2 cm below the soil surface. The CL was determined from the scutellum to the tip of the coleoptile.

### SNP genotyping and QTL analysis

For the Zhou 8425B × Chinese Spring population, the 245 RILs and their parents were genotyped with the 90K iSelect SNP array (Wang et al., 2014). Twenty-one linkage groups corresponding to the 21 chromosomes were constructed from 14,955 polymorphic markers. All linkage maps covered 2290.06 cM with marker densities of 7.04 (A), 8.60 (B) and 2.19 (D) markers per cM (Wen et al., 2017). Broad-sense heritability was estimated using IciMapping 4.1 software (https://isbreeding.caas.cn/index.htm). Quantitative trait loci (QTLs) mapping was conducted using IciMapping 4.1 software with inclusive composite interval mapping (ICIM) algorithm (Li et al., 2007). The CL of all lines and the average phenotypic values from the two environments were used for QTL detection. The mapping parameters were chosen as step=1.0 cM and PIN = 0.01. A LOD threshold of 2.5 was chosen for declaration of putative QTLs.

### Plant materials for *Rht-B1* functional study and RNA-Seq analysis

To study the association between *Rht-B1* and the 4B QTL in the current study, an overexpression line and loss of function line of *Rht-B1* were created. The complete coding sequence (CDS) of *Rht-B1b* (GenBank: MG681100.1) was overexpressed in a hexaploid wheat cultivar Fielder (*Rht-B1b* and *Rht-D1a*) under driving by maize ubiquitin promoter (All primers were listed in **Table S1**). CRISPR/SpCas9 was used to create knockout line of *Rht-B1b*. The sgRNA (PAM-guide sequence 5’-GGAGCCGTTCATGCTGCAG-3’) was designed to target conserved regions of *Rht-B1b*. The resultant construct was transformed into immature embryos by the *Agrobacterium tumefaciens* (Ishida et al., 2015). Sixty good-quality seeds of each transgenic null lines (TNL), *Rht-B1b* overexpression lines (*Rht-B1b*-OE) and *Rht-B1b* CRISPR/SpCas9 edited lines (*Rht-B1b*-KO) were evenly sown in ten 10 cm (top diameter) × 8.9 cm (height) plant pots with a sowing depth of 2 cm below the soil surface. Before the coleoptile broke the ground, coleoptile tips and whole coleoptiles of ten TNL and *Rht-B1b*-OE plants were collected and immediately put into liquid nitrogen for RNA-Seq. Each group included three biological replicates.

### RNA-Seq and data analysis

Total RNA of three biological replicates was extracted using the TRIzol^®^ reagent, and mRNA was purified from total RNA using poly-T oligo-attached magnetic beads. The first strand cDNA was synthesized using random hexamer primer and RNase H. Subsequently, the second strand cDNA synthesis was obtained using DNA Polymerase I and RNase H. Library preparation for RNA-Seq was conducted by Novogene and sequenced on an Illumina Novaseq platform with 1 ug of total RNA (http://www.novogene.com/).

IWGSC RefSeq v2.1 and annotation v2.1 were used for the reference genome and gene model annotation (Zhu et al., 2021). Raw data were processed to obtain clean reads by removal of adapter, ploy-N and low-quality reads. Paired-end clean reads were aligned to the reference genome using Hisat2 (Kim et al., 2019). FeatureCounts was used to count the read numbers mapped to each gene (Liao et al., 2014). Differential expression analysis was performed using the DESeq2 R package (Love et al., 2014). Genes with an adjusted *P*-value < 0.05 found by DESeq2 were assigned as differentially expressed genes (DEG). GO and KEGG enrichment analysis of DEGs were implemented by the TBtools (Chen et al., 2020). GO terms and KEGG pathways with corrected *P*-value lower than 0.05 were considered as significantly enriched by the DEGs.

## Results

### QTL analysis of coleoptile length

**Figure 1:**
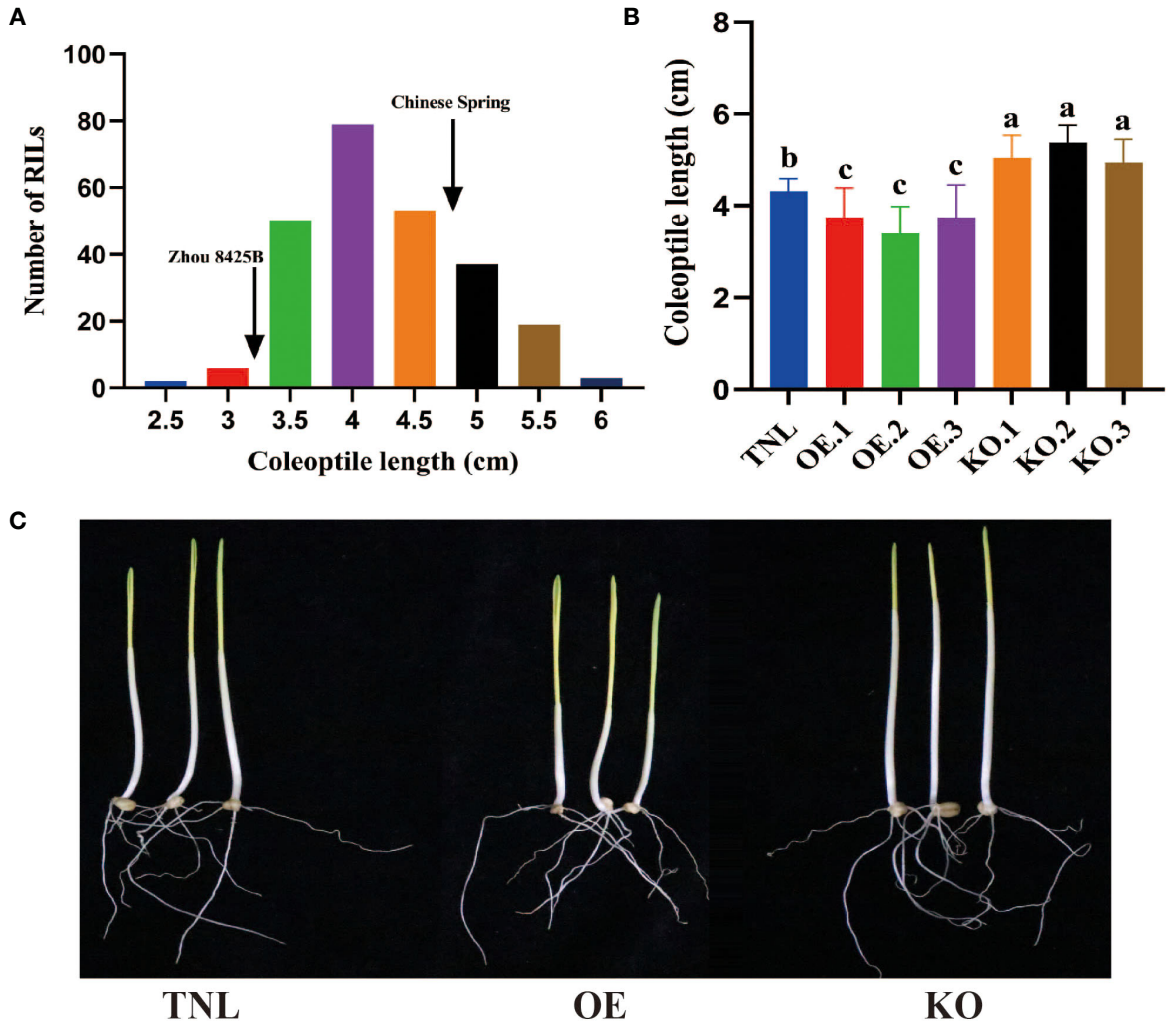
Frequency distributions of RILs for CL and effects of *Rht-B1b* on coleoptiles of wheat. **(A)** Frequency distributions of 245 recombinant inbred lines (RILs) in Zhou 8425B × Chinese Spring population for mean values of coleoptile length (CL). Arrows indicate mean values of the parental lines. Coleoptile length **(B)** and image of seedlings **(C)** of *Rht-B1b* overexpressing lines (OE), *Rht-B1b* knockout lines (KO) and transgenic null lines (TNL). Bars represent standard deviations of thirty biological replicates. Different letters on the bars indicate significant differences in given traits at *P* < 0.05 between different lines.

The parental lines, Zhou 8425B and Chinese Spring, differed significantly (*P* < 0.05) for coleoptile length (CL). Based on data averaged across all environments, CL ranged from 2.5 to 6.1 cm with an average of 4.0 cm. CL showed continuous variation in RIL population and had a high heritability of 0.86 (**Figure 1A**). Three QTLs for CL were identified on chromosomes 2BL, 4BS and 4DS in the Zhou 8425B × Chinese Spring population (**Table 1**). Two major QTLs, *QCL.qau-4BS* and *QCL.qau-4DS*, were stably detected in all environments, which explained 9.1%–22.2% of the phenotypic variance across environments (**Table 1**). Based on the genomic position of the flanking markers, we found that *QCL.qau-4BS* and *QCL.qau-4DS* spanned the *Rht-B1* and *Rht-D1* loci, respectively. *QCL.qau-2BL* explained about 3.0%–3.1% of phenotypic variance, and thus was a minor QTL for CL.

**Table 1:** QTL for coleoptile length (CL) in the Zhou 8425B/Chinese Spring RIL population.

Envi^a^	QTL	Pos (cM)	Marker interval	Pos (Mb)	LOD	PVE (%)	Add^b^
CL1	*QCL.qau-4BS*	24	*IWB24098*-*IWB56078*	26.4-34.9	17.9	22.2	-0.3
CL1	*QCL.qau-4DS*	17	*IWB53820*-*IWB8050*	14.1-32.9	8.6	10	-0.2
CL2	*QCL.qau-2BL*	167	*IWB5439*-*IWB66206*	638.6-784.5	2.9	3	0.1
CL2	*QCL.qau-4BS*	24	*IWB24098*-*IWB56078*	26.4-34.9	16.7	18.4	-0.3
CL2	*QCL.qau-4DS*	18	*IWB8050*-*IWA7344*	32.9-50.6	8.4	9.1	-0.2
CL	*QCL.qau-2BL*	167	*IWB5439*-*IWB66206*	638.6-784.5	3.2	3.1	0.1
CL	*QCL.qau-4BS*	24	*IWB24098*-*IWB56078*	26.4-34.9	19.7	21.1	-0.3
CL	*QCL.qau-4DS*	16	*IWB53820*-*IWB8050*	14.1-32.9	11	13.2	-0.2

a CL1, CL2 and CL indicated that phenotypic data were collected from Shijiazhuang in 2021, Qingdao in 2022 and average phenotypic data.

b Negative “additive effect” indicates an increasing effect from Chinese Spring; positive “additive effect” indicates an increasing effect from Zhou 8425B.

### Validation of the effect of *Rht-B1b* on coleoptile development

Many studies have reported CL QTL on the *Rht-B1* locus, indicating that *Rht-B1b* may reduce wheat CL (Botwright et al., 2001; Li et al., 2017). However, there has been no direct evidence for this speculation. Here, *Rht-B1b* overexpression and CRISPR/SpCas9 gene-editing were performed and homozygous plants were generated by self-crossing for CL evaluation. The results demonstrated that *Rht-B1b* overexpression could reduce the CL about 8.6%, while loss-of-function of *Rht-B1b* would increase the CL about 17.9% relative to that of null transgenic plants (TNL) (**Figures 1B, C**). Thus, *Rht-B1* can be a target gene of *QCL.qau-4BS*.

### Transcriptome analysis of *Rht-B1* on coleoptile development

Although *Rht-B1b* is known to reduce the CL, the underlying regulatory mechanism remains unclear. To dissect the regulatory mechanism, whole coleoptiles and coleoptile tips of TNL and *Rht-B1b*-OE were collected for RNA-Seq analysis before the coleoptile breaks the ground. Compared with those of TNL, 142/523 and 191/1993 differentially expressed genes (DEG) were upregulated/downregulated by *Rht-B1b* in the transcriptome of whole coleoptile and coleoptile tips (**Table S2 and S3**). There were more down-regulated DEGs than up-regulated DEGs, indicating that *Rht-B1b* mainly represses the gene expression in coleoptiles. GO enrichment analysis of coleoptile tips revealed that *Rht-B1b* mainly reduces the CL *via* the process of “photosynthesis”, “oxidation-reduction process”, “nitrate assimilation carbohydrate metabolic process”, and “pigment biosynthetic process”. In the whole coleoptile, DEGs were enriched in the GO processes of “oxidation-reduction”, “glucan metabolism” and “cellular carbohydrate metabolism” (**Table 2**). In the coleoptile tips, DEGs were mainly enriched in the GO processes of “photosynthesis”, “oxidation-reduction”, “nitrate assimilation”, “carbohydrate metabolism” and “pigment biosynthetic process” (**Table 2**).

**Table 2:** Enrichment analysis of the most significant GO processes in the transcriptome of coleoptile tips and whole coleoptiles.

Groups^a^	GO_ID	GO_Name	DEG^b^	FDR^c^
Tip	GO:0015979	photosynthesis	67	0
Tip	GO:0055114	oxidation-reduction process	214	3.80E-10
Tip	GO:0042128	nitrate assimilation	5	0.0020535
Tip	GO:0005975	carbohydrate metabolic process	71	0.0175129
Tip	GO:0046148	pigment biosynthetic process	5	0.0172859
Coleoptile	GO:0055114	oxidation-reduction process	94	1.55E-10
Coleoptile	GO:0044042	glucan metabolic process	12	6.08E-04
Coleoptile	GO:0044262	cellular carbohydrate metabolic process	13	0.0012301

a Tip and Coleoptile represent coleoptile tip and whole coleoptile.

b DEG stands for Differentially expressed genes.

c FDR stands for False Discovery Rate. FDR < 0.05 represents the DEG were significantly enriched in the GO process.

**Figure 2:**
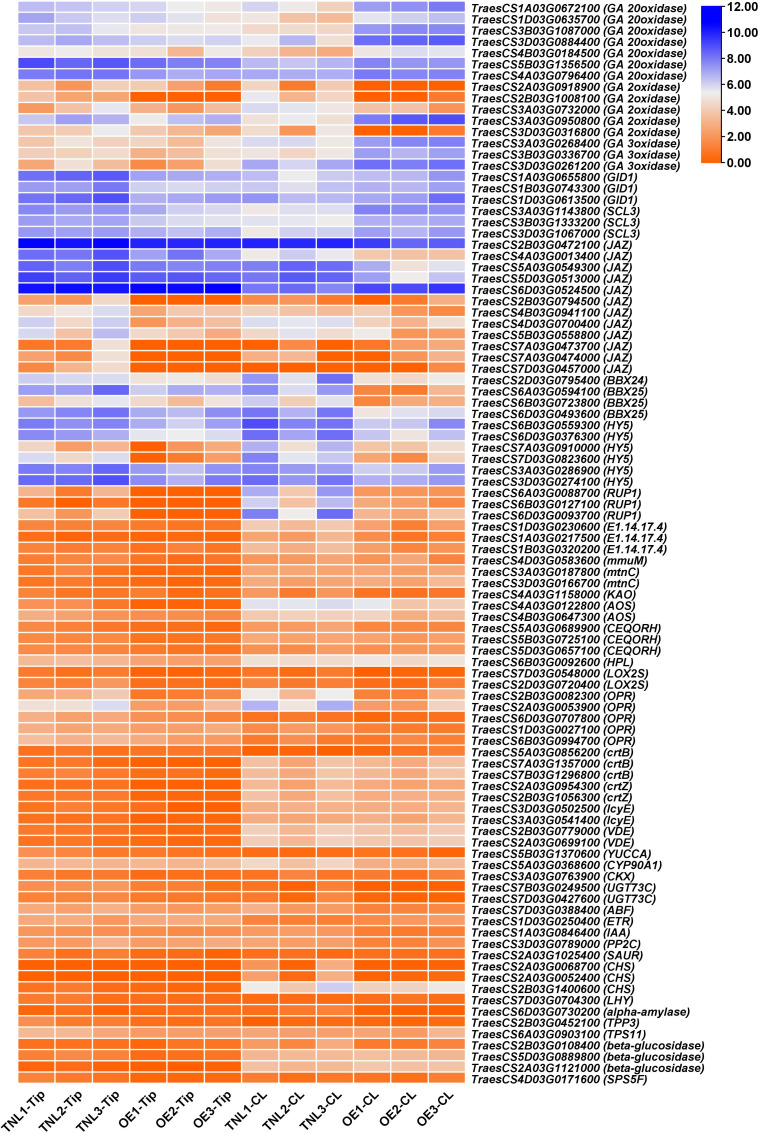
Putative key downstream genes of *Rht-B1b* for coleoptile development. TNL, OE, Tip and CL represents transgenic null plant, *Rht-B1b*-OE plant, coleoptile tip and whole coleoptile, respectively. Orange and blue colors show genes with low and high expression level, respectively.

Previous studies showed that hypocotyl elongation is regulated by endogenous regulators, such as phytohormones, circadian clock, sucrose, and environmental stimuli (Saibo et al., 2003; Simon et al., 2018). Interestingly, many DEGs were enriched in the KEGG pathway of plant hormone signal transduction, alpha-linolenic acid metabolism (jasmonic acid), brassinosteroid biosynthesis, carotenoid biosynthesis (abscisic acid), cysteine and methionine metabolism (ethylene), diterpenoid biosynthesis (gibberellin), tryptophan metabolism (auxin), zeatin biosynthesis (cytokinine), circadian rhythm, and starch and sucrose metabolism (**Figure 2**). Thus, *Rht-B1b* might play an important role in integrating multiple signal transduction pathways in the wheat coleoptile development.

## Discussion

### Adaptation of semi-dwarf modern wheat to drought conditions

Numerous studies have demonstrated a positive association between wheat CL and plant number under deep sowing (Hadjichristodoulou et al., 1977; Matsui et al., 2002). However, the two gibberellin-insensitive dwarfing genes, *Rht-B1b* and *Rht-D1b*, tend to cause shorter CL and low seedling emergence rate (Schillinger et al., 1998; Rebetzke et al., 2007b). Here, we generated *Rht-B1b* over-expressing and CRISPR/SpCas9 editing plants to study its influence on coleoptile development. As a result, overexpression of *Rht-B1b* reduced the CL, while its loss of function increased the CL. *Rht-B1b* encodes an N-terminal truncated DELLA protein (lack of DELLA and TVHYNP motifs), which is gibberellin-insensitive protein in wheat (Van De Velde et al., 2021). DELLA proteins encoded by the *Rht-B1a* gene are the downstream repressors of GA signal transduction and, GA induces the degradation of DELLA proteins *via* the ubiquitin/proteasome pathway (Itoh et al., 2003). Thus, *Rht-B1b* led to a reduction of CL compared with tall allele *Rht-B1a* since the GA-induced seedling growth was repressed (Alabadií et al., 2004). So far, there has been no study to validate the effect of *Rht-B1b* on CL, not to mention the underlying genetic pathway. To uncover the regulatory mechanism of *Rht-B1b* on CL, a transcriptome analysis was conducted to dissect the underlying genetic pathway.

**Figure 3:**
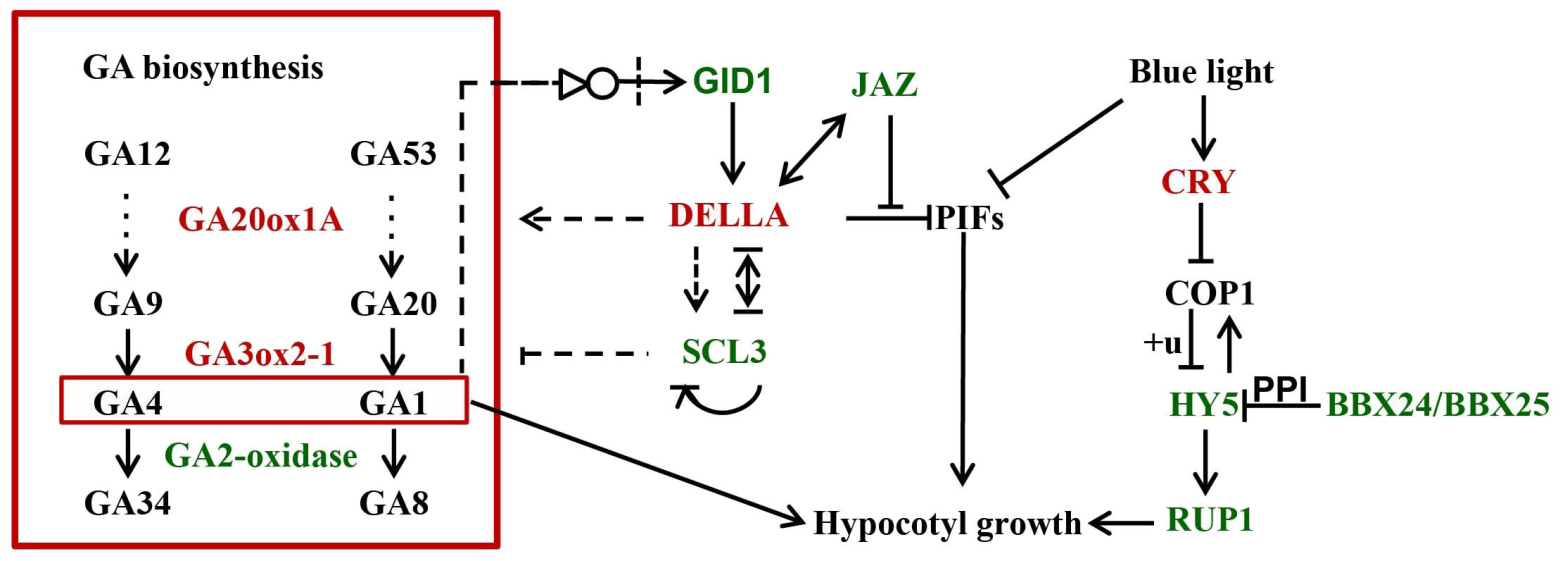
A simplified model underpinning *Rht-B1b* modulating coleoptile length. Black letters in the box show non-differentially expressed genes. Red and green letters show upregulated and downregulated genes by *Rht-B1b*, respectively. The +u represents ubiquitylation. GA represent gibberellin. The arrows show promotion of gene expression; the lines with blunt ends show repression of gene expression; the bold lines represent direct binding. The species latin prefixes in gene names are not shown.

A few studies have demonstrated that, in *Arabidopsis*, SCL3 and DELLA antagonize each other in modulating downstream GA responses and maintaining GA homeostasis *via* feedback regulation of GA biosynthetic genes (Zhang et al., 2011). SCL3 functions as a positive regulator of GA signaling, which induces the expression of GA biosynthesis genes and autoregulates its own expression *via* direct interaction with DELLA. In our transcriptome, 16 genes related to GA biosynthesis, three GA receptor GID1 genes and three SCL3 genes were identified as DEGs between *Rht-B1b*-OE and TNL, indicating that wheat SCL3 and DELLA antagonize each other in maintaining GA homeostasis and GA responses as in *Arabidopsis* (**Figure 2**, **3**). In addition, DELLAs can physically interact with and block PIF3 and PIF4 activities by sequestering the transcription factors from binding to their targets, which ultimately results in the inhibition of hypocotyl elongation (De lucas et al., 2008; Feng et al., 2008). JAZ could interrupt DELLA–PIF3 interaction, allowing more PIF transcription factors to activate plant growth (Yang et al., 2012). In our transcriptome, several homologs of JAZs were down-regulated by *Rht-B1b*-OE (**Figure 2**, **3**). HY5 is a key transcription factor for the regulation of seedling photomorphogenesis. COP1 negatively regulates HY5 by directly and specifically interacting with HY5 (Ang et al., 1998). BBX25 and BBX24 additively enhance COP1 and suppress HY5 functions to regulate of seedling deetiolation process in *Arabidopsis* (Gangappa et al., 2013). *RUP1* is induced by CRYs in response to blue light, which is dependent on HY5 (Tissot and Ulm, 2020). These genes are key factors for cryptochrome blue-light signaling and their homologs in wheat were identified as DEGs, indicating that light and GAs might antagonistically regulate coleoptile in wheat (**Figure 2**, **3**).

### Breeding for longer coleoptiles with previously reported genetic loci

**Figure 4:**
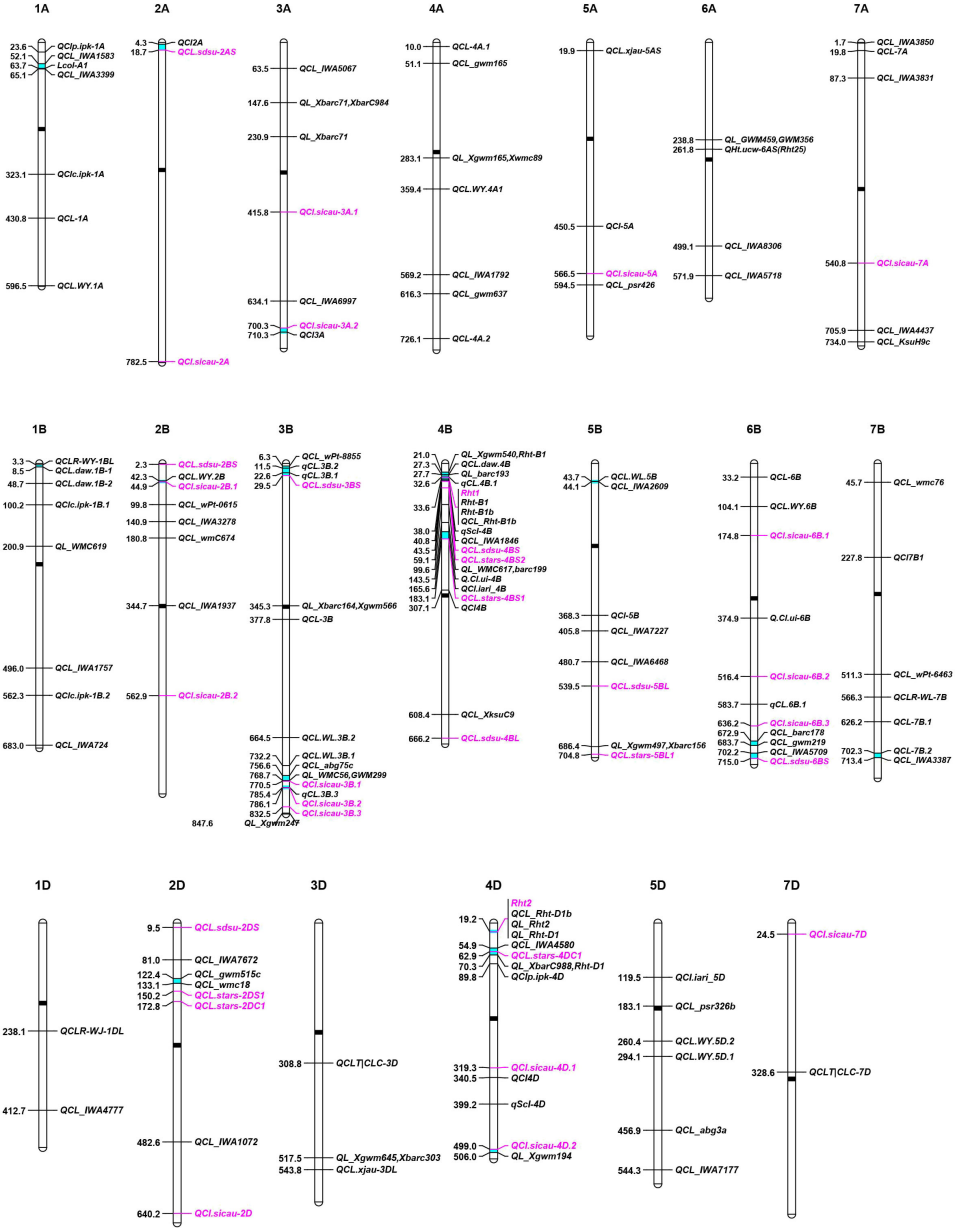
Distribution of genetic loci for wheat coleoptile length (CL) on chromosomes. QTLs and GWAS loci are indicated in black and pink colors, respectively. The black and cyan bars in chromosomes indicate the positions of centromeres and QTL-rich clusters (QRC), respectively.

The response of plants to drought is dependent on multiple factors, including duration and severity of drought conditions, frequency of drought, and the growth stage when subjected to the drought stress (Jatayev et al., 2020). Although wheat can be grown in a variety of harsh environments, rising temperature and unpredictable drought exacerbate the impact of drought stress on wheat yield. If drought stress occurs when sowing, farmers tend to sow more seeds in a deeper depth to increase the seedling establishment rate. Short coleoptiles severely hider the application of deep sowing in wheat production since it influences the emergence rate of wheat seedlings, particularly in fields with thick stubble or crusted soil surface (Rebetzke et al., 2014). Most modern semi-dwarf wheat varieties harboring *Rht-B1b* or *Rht-D1b* have short coleoptiles and low yields under drought stress relative to tall plants (Li et al., 2017; Sidhu et al., 2020). Wheat CL is a typical quantitative trait controlled by multiple genes (Rebetzke et al., 2007a). Pyramiding of multiple QTLs in modern semi-dwarf wheat cultivars can efficiently increase the CL. Thus, a comprehensive screening was conducted on the genetic locus for CL by QTL mapping and genome wide association analysis (GWAS) from previous studies and assembled them on wheat chromosomes according to their physical locations (**Figure 4**). So far, a total of 114 QTLs for CL traits in wheat have been found from 20 studies of CL-related QTL mapping in wheat (**Figure 4** and **Table S4**) (Rebetzke et al., 2001; Rebetzke et al., 2007b; Landjeva et al., 2008; Landjeva et al., 2010; Li et al., 2010; Yu and Bai, 2010; Li et al., 2011; Zhang et al., 2013; Nagel et al., 2014; Rebetzke et al., 2014; Zhang et al., 2014; Singh et al., 2015; Elbudony, 2017; Liu et al., 2017; Mo et al., 2018; Zhang et al., 2018; Bovill et al., 2019; Puttamadanayaka et al., 2020; Francki et al., 2021; Ren et al., 2021). About 33 GWAS loci were found to be associated with CL (**Figure 4** and **Table S5**) (Li et al., 2017; Ma et al., 2020; Sidhu et al., 2020). These genetic loci were used for QTL-rich cluster (QRC) detection, which was defined when markers from at least two independent studies were physically located in 10 Mb range (Cao et al., 2020). The genomic positions of flanking markers were obtained from the IWGSC RefSeq V2.1 (Zhu et al., 2021). A total of 18 QTL-rich clusters (QRC) for CL were found in this study (**Table 3**). Of them, *Rht-B1* and *Rht-D1* are strong candidate genes of QRC 4B-I and 4D-I, respectively. These QRC of CL provide valuable gene resources for marker-assisted selection breeding for longer coleoptiles.

**Table 3:** Detailed information and candidate genes for QTL-rich clusters of coleoptile length.

QRC	Interval (Mb)^a^	CL Locus	Candidate genes
1A-I	52.1-65.1	*QCL_IWA1583*; *Lcol-A1*; *QCL_IWA3399*	
1B-I	3.3-8.5	*QCLR-WY-1BL*; *QCL.daw.1B-1*	
2A-I	4.2-18.6	*QCl2A*; *QCL.sdsu-2AS*	
2B-I	42.2-44.9	*QCL.WY.2B*; *QCl.sicau-2B.1*	
2D-I	122.3-133.1	*QCL_gwm515c*; *QCL_wmc18*	
3A-I	700.2-710.2	*QCl.sicau-3A.2*; *QCl3A*	
3B-I	6.29-29.4	*QCL_wPt-8855*; *qCL.3B.2*; *qCL.3B.1*; *QCL.sdsu-3BS*	
3B-II	756.5-770.4	*QCL_abg75c*; *QL_WMC56, GWM299*; *QCl.sicau-3B.1*	
3B-III	785.4-786.0	*qCL.3B.3*; *QCl.sicau-3B.2*	
4B-I	21.0-43.5	*QL_Xgwm540*; *QCL.daw.4B*; *QL_barc193*; *qCL.4B.1*; *Rht1*; *Rht-B1*; *Rht-B1b*; *QCL_Rht-B1b*; *qScl-4B*; *QCL_IWA1846*; *QCL.sdsu-4BS*	*Rht-B1*
4B-II	165.6-183.0	*QCl.iari_4B*; *QCL.stars-4BS1; QCL.qau-4BS*	
4D-I	19.1	*Rht2*; *QCL_Rht-D1b*; *QL_Rht2*; *QL_Rht-D1; QCL.qau-4DS*	*Rht-D1*
4D-II	54.9-70.3	*QCL_IWA4580*; *QCL.stars-4DC1*; *QL_Xbarc288*	
4D-III	498.9-505.9	*QCl.sicau-4D.2*; *QL_Xgwm194*	
5B-I	43.6-44.0	*QCL.WL.5B*; *QCL_IWA2609*	
6B-I	672.9-683.7	*QCL_barc178*; *QCL_gwm219*	
6B-II	702.1-714.9	*QCL_IWA5709*; *QCL.sdsu-6B*	
7B-I	702.2-713.4	*QCL-7B.2*; *QCL_IWA3387*	

a The intervals of QTL-rich clusters (QRC) were defined according to IWGSC RefSeq v2.1.

### Breeding for longer coleoptiles with the wild allele *Rht-B1a*

In the 1960s and 1970s, the wide application of semi-dwarf genes *Rht-B1b* and *Rht-D1b* combined with the increased application of chemical fertilizer greatly promoted the wheat yield improvement, which was referred to as the “Green Revolution” of wheat (Hedden, 2003). However, semi-dwarf wheat with *Rht-B1b*/*Rht-D1b* alleles produce lower grain yield than taller plants with *Rht-B1a*/*Rht-D1a* under drought environment (Zhang et al., 2013; Jatayev et al., 2020). Compared with wild-type *Rht-B1a*, semi-dwarf allele *Rht-B1b* resulted in shorter coleoptile. Seeds of semi-dwarf wheat cultivars are generally sown shallower than taller wheat varieties to ensure the emergence of semi-dwarf seedlings and early vigour (Rebetzke et al., 2007a; Jatayev et al., 2020). Shallow seeding in dry fields reduces emergence for varieties with short coleoptile length (Rebetzke et al., 2001; Jatayev et al., 2020). It is likely that taller wheat cultivars with *Rht-B1a*/*Rht-D1a* have higher rate of emergence than semi-dwarf wheat genotypes under early drought environment. Thus, improving lodging resistance of tall wheat has become an important research direction. Besides of reducing the height of plants, an alternative way is to breed wheat varieties with solid-stemmed stems to enhance lodging resistance in wheat (Liang et al., 2022). Wheat with tall plant height *Rht-B1a*/*Rht-D1a* allele combined with solid-stemmed stems alleles of *TdDof* might have high lodging and drought tolerances, long coleoptile and produce higher yield in drought field (Jatayev et al., 2020; Nilsen et al., 2020).

## Data availability statement

The data presented in the study are deposited in the Sequence Read Archive (SRA) of the National Center for Biotechnology Information in the BioProject with accession number PRJNA936995.

## Author contributions

DX: Project administration, Investigation, Visualization, Writing, Editing and Funding acquisition. QH: Investigation, Data curation, Writing. TY: Investigation, Data curation. XL: Investigation, Data curation. HQ: Investigation. YW: Investigation. CJ: Investigation. WL: Investigation. XD: Investigation. JZ: Investigation. HZ: Investigation. ZH: Resources XX: Resources. SC: Review, Supervision and Resources. WM: Review, Supervision, and Funding acquisition. All authors contributed to the article and approved the submitted version.
